# The Association of Environmental Variables with Intelligence and Well-Being in Copy Number Variation (CNV) Carriers

**DOI:** 10.21203/rs.3.rs-8611446/v1

**Published:** 2026-02-10

**Authors:** Yelyzaveta Snihirova, Sebastien Jacquemont, Gabriëlla Blokland, Sinan Guloksuz, Therese Amelsvoort van, David Linden

**Affiliations:** Maastricht University; Université de Montréal; Department of Psychiatry and Neuropsychology, Faculty of Health, Medicine, and Life Sciences, Maastricht University, Maastricht, the Netherlands; Maastricht University; Maastricht University; Maastricht University

## Abstract

Copy Number Variants (CNVs) are structural genomic alterations that have been linked to cognitive deficits and susceptibility to neurodevelopmental and psychiatric disorders, with their impact often shaped by interactions with environmental factors such as socioeconomic status (SES), stress, and lifestyle. This study leverages UK Biobank data to explore how these interactions influence fluid intelligence and well-being, two key indicators of cognitive and psychosocial functioning, in CNV carriers, aiming to identify factors for personalized interventions. We investigated interactions between common pathological CNVs – 1q21.1 distal, 15q11.2, 15q13.3 BP4–BP5, 16p11.2 proximal and distal, and 22q11.2 proximal and distal – and key environmental variables, including SES, stress, and lifestyle, on fluid intelligence and well-being. Using ANOVA models with interaction terms, we examined how interactions between CNV status and environmental factors impact cognition and well-being. SES variables interacted with CNV status in distinct ways. While non-carriers exhibited the expected association of higher fluid intelligence with increasing SES, 16p11.2 distal deletion carriers in the moderate SES group had lower fluid intelligence than both non-carriers in the same SES group and carriers with low SES. Stress-related interactions varied by CNV type: in 16p11.2 proximal duplication carriers, higher adulthood stress was significantly associated with increased fluid intelligence. Furthermore, 15q11.2 deletion carriers exhibited significantly lower fluid intelligence in the moderately healthy lifestyle group compared to all non-carrier groups (p < 0.01). By revealing how environmental factors interact with CNV status, this study identifies potential targets for personalized interventions aimed at supporting cognitive and emotional resilience in genetically at-risk individuals.

## Introduction

Copy Number Variants (CNVs) are structural genomic alterations that contribute to genetic diversity and disease susceptibility^1–3^. Pathogenic CNVs are strongly associated with cognitive impairments, affecting memory, executive function, and problem-solving, and increasing vulnerability to neurodevelopmental and psychiatric disorders^4–9^. Even asymptomatic CNV carriers often exhibit subtle but measurable cognitive deficits that impact daily functioning and quality of life^10,11^.

Genetic influences on cognition do not act in isolation. Environmental factors such as socioeconomic status (SES), education, and stress interact with genetic predispositions, particularly during early development^12–14^. Individuals genetically predisposed to higher cognitive function may benefit more from enriched environments^15^, while adverse conditions can disproportionately impair cognition^16–18^. This aligns with the differential susceptibility model, which proposes that genetically vulnerable individuals are more sensitive to both positive and negative environmental influences. CNV carriers, for example, may experience amplified cognitive difficulties in high-stress environments but show improvement with tailored educational or therapeutic support^19,20^.

Most CNVs are associated with lower cognitive functioning, reducing educational attainment and income potential. Impairments caused by CNVs hinder academic and professional success and limit individuals’ ability to adapt to complex social or occupational environments, compounding stress and reducing psychological resilience. As a result, the interplay between cognitive challenges associated with CNVs and environmental factors, such as socioeconomic status or educational access, can exacerbate disadvantages, leading to negative outcomes in mental health and quality of life^10,11^. However, how environmental influences interact with CNVs to affect fluid intelligence – the capacity to solve novel problems and adapt through reasoning and working memory^21^ – and well-being, remains poorly understood and warrants further investigation.

The UK Biobank provides a valuable resource for investigating how SES, stress, and lifestyle interact with CNVs to influence fluid intelligence and well-being. Understanding how environmental factors shape cognition and well-being can offer a more holistic basis for personalized interventions. We aim to compare CNV carriers with non-carriers to identify environmental factors that may mitigate or amplify the impact of CNVs on cognition and well-being. Understanding these interactions could inform clinical strategies for early identification of at-risk individuals and guide interventions to optimize cognitive function and life satisfaction through targeted environmental support. These insights could contribute to personalized approaches to improve the quality of life and resilience in CNV carriers.

## Materials and methods

### Participants

The UK Biobank recruited ~ 500 000 participants (54% female; aged 40–69) between 2006 and 2010 for cognitive, phenotypic, and health assessments, with follow-ups conducted in person and online. Participants provided written informed consent, and the study was approved by the North West MultiCentre Ethics Committee (11/NW/0382). Recruitment was via NHS registers without exclusion criteria. Data were accessed under application 55392. Ethnicity was self-reported (Data-Field: 21000) and classified as White (British, Irish, or other) or Non-White for covariate use.

### CNV calling and annotation

CNVs were identified using PennCNV and QuantiSNP following a published pipeline^22,23^, with detection thresholds of ≥ 3 consecutive probes, ≥ 1 kb size, and confidence scores ≥ 15. The DigCNV package merged and filtered CNVs across algorithms (https://github.com/labjacquemont/DigCNV). CNVs were retained if they had a confidence score ≥ 30 in at least one algorithm, size ≥ 50 kb, clear type (deletion/duplication), and < 50% overlap with segmental duplications, HLA, or centromeres. Each CNV had to cover ≥ 40% of a known pathogenic region (hg19), focusing on recurrent variants well represented in the UK Biobank^24^.

### Fluid Intelligence

Fluid intelligence (Data-Field: 20016) was assessed using a touchscreen questionnaire with 13 timed questions. Scores ranged from 0 to 13 based on correct answers, with unanswered questions scored as zero.

### Well-being

A principal component analysis (PCA) was conducted on 5 well-being-related items (Data-Fields: 4526, 4548, 4559, 4570, 4581) covering happiness and satisfaction with family, friendships, health, and finances to derive a composite index. Before analysis, responses were coded so that a higher score indicated a higher level of well-being. The first principal component was retained, accounting for 48% of the total variance. This component was extracted as the well-being index, with higher scores indicating greater well-being^25^.

### Environmental measures

#### Socioeconomic status

SES was derived by latent class analysis using family income, occupation or employment status, and education level, and three levels (low, medium, and high) were defined according to item response probabilities^26^. We used the three-latent-class solution as described by the authors. Three groups according to SES were generated: “Low SES”, “Medium SES”, and “High SES” according to the factor-response probabilities.

Separately from the SES score, we used the Townsend deprivation index, household income, educational level, and employment status as individual independent factors^27^.

The Townsend deprivation index (Data-Field: 189) equates to levels of socioeconomic deprivation; however, we reversed the scale for consistency (higher score is better), and we referred to this as the “Townsend deprivation index (reversed)” or “wealth”.

#### Stress

We calculated two stress categories: childhood stress (CS) and adulthood stress (AS), following the previously described method^28^. CS was assessed using five questions (Data-Fields: 20487–20491) relating to how well-loved and looked after participants were as children, each answered on a five-point rating scale with reverse coding where necessary. These questions were based on a shortened version of the Childhood Trauma Questionnaire (CTS-5), a modified version of an established and widely used measure of adverse life experiences occurring during childhood. AS was measured using a similar set of five questions (Data-Fields: 20521–20525) relating to the experience of potentially abusive relationships experienced as an adult, also using a five-point scale. Total CS and AS scores were calculated as the sum of these five questions, respectively.

#### Lifestyle

A lifestyle score was constructed using a previously established method^29^, incorporating ten UK Biobank variables: smoking, alcohol use, physical activity, sedentary time, sleep duration, and dietary factors (fruit/vegetables, oily fish, red meat, processed meat). Each unhealthy behavior received one point (e.g., smoking, < 150 min/week activity, etc.). A composite lifestyle score was then calculated for each participant by summing the total number of unhealthy lifestyle factors, ranging from 0 to 9. Based on the composite score, participants were classified into three lifestyle categories: most healthy (0–2), moderately healthy (3–5), and least healthy (6–9).

#### Statistical analyzes

Analyses were performed in R v4.4.1^30^. Missing responses (e.g., “do not know”) were treated as missing values.

We conducted ANOVA models testing interactions between CNV status (carrier vs. non-carrier) and environmental factors (SES, Townsend index, income, education, work status, adult and childhood stress, lifestyle) on fluid intelligence and well-being. Each model included age, sex, and ethnicity as covariates, and individuals with other CNVs were excluded. A total of 112 models were run for each output, with False Discovery Rate (FDR) correction applied within each trait domain. Post hoc comparisons were conducted using estimated marginal means with Tukey adjustment to evaluate group differences.

## Results

### Description of CNV carriers

After applying quality control filters, 502 133 UK Biobank participants were included in the analysis (Table 1). We found some sex differences in CNV prevalence: 15q13.3 BP4–BP5 (*CHRNA7*) duplications were more common in males (χ^2^(1) = 33.109, p < 0.001; 146 vs. 79 carriers), while 15q11.2 deletions were more frequent in females (χ^2^(1) = 6.78, p = 0.009; 682 vs. 659 carriers). Carriers of 16p11.2 proximal (p < 0.001) and 22q11.2 proximal deletions (p = 0.02) were substantially younger than noncarriers (Table S1).

Significant differences in FI were observed for multiple CNVs, including 1q21.1 distal deletions (p = 0.03), 15q11.2 deletions (p < 0.001), 15q13.3 BP4–BP5 (*CHRNA7*) duplications (p < 0.001), 16p11.2 proximal duplications (p < 0.001) and distal deletion (p < 0.001), and 22q11.2 proximal deletions (p = 0.04) and duplications (p < 0.001), with carriers consistently exhibiting lower FI scores compared to non-carriers. Significant differences in well-being were identified for multiple CNVs. For example, the 15q13.3 BP4–BP5 (*CHRNA7*) deletion (p = 0.02), both duplications and deletions of the 16p11.2 proximal region (p = 0.01, p = 0.03), the 16p11.2 distal deletion (p = 0.004), and the 22q11.2 proximal duplication (p = 0.02) were all associated with lower well-being scores in carriers compared to non-carriers (Table S2).

Within the predominantly European ancestry UK Biobank cohort, the 15q11.2 deletion (p < 0.001) and the 15q13.3 BP4–BP5 (*CHRNA7*) duplication (p = 0.03), along with the 16p11.2 and 22q11.2 proximal duplications (p = 0.04 and p = 0.02, respectively), show significant differences in frequency, with these CNVs being more common among individuals of European descent (Table S1). These findings can reflect recruitment bias and the demographic composition of the cohort. The tables presenting differences in sex, ethnicity, and age across CNVs, as well as comparisons of fluid intelligence and well-being between carriers and non-carriers, are provided in the Supplementary Materials.

### Socioeconomic status (SES)

**Figure 1:**
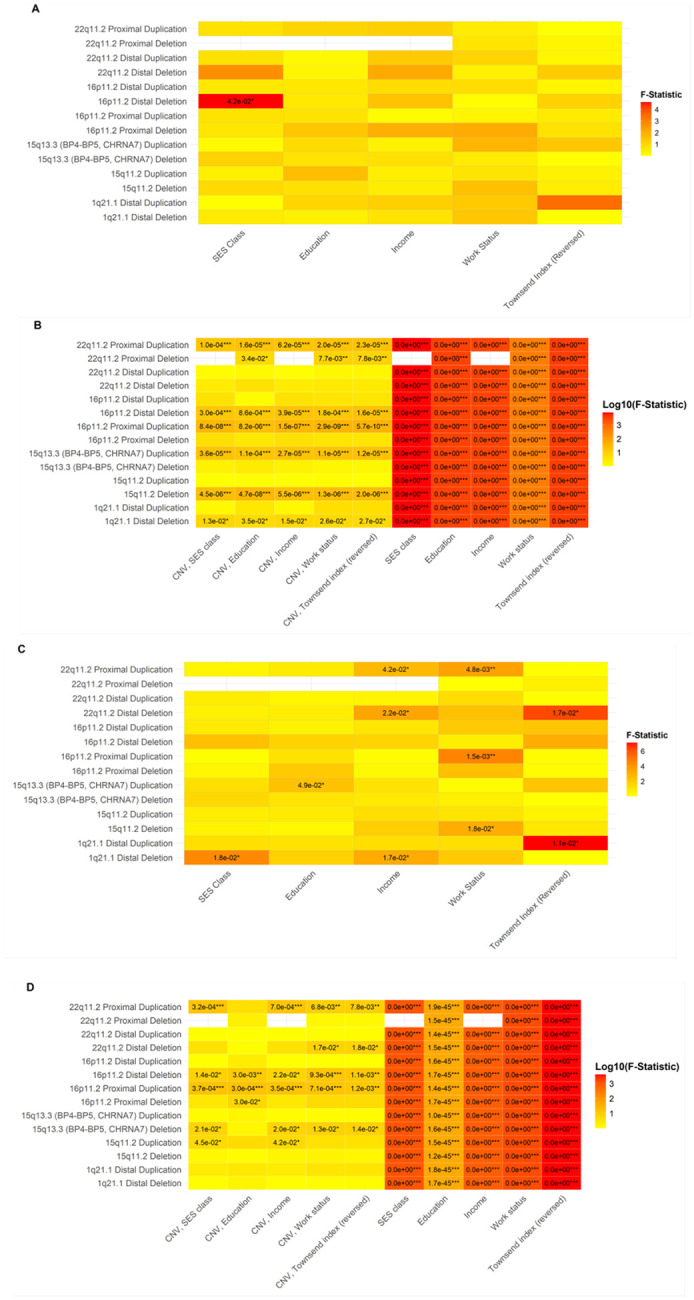
Heatmaps from ANOVAs illustrating the effects of socioeconomic status (SES) and Copy Number Variants (CNVs) on fluid intelligence and well-being. (A, C) Interaction effects: SES measures (x-axis) versus CNVs (y-axis) with colour-coded F-statistics and numeric p-values for fluid intelligence (A) and well-being (C). (B, D) Main effects: Same axes with a colour scale representing log_10_-transformed p-values for fluid intelligence (B) and well-being (D). “CNV, X” indicates the main effect of CNV status in the ANOVA model predicting environmental variable X (e.g., education, income, etc.).

**Figure 2:**
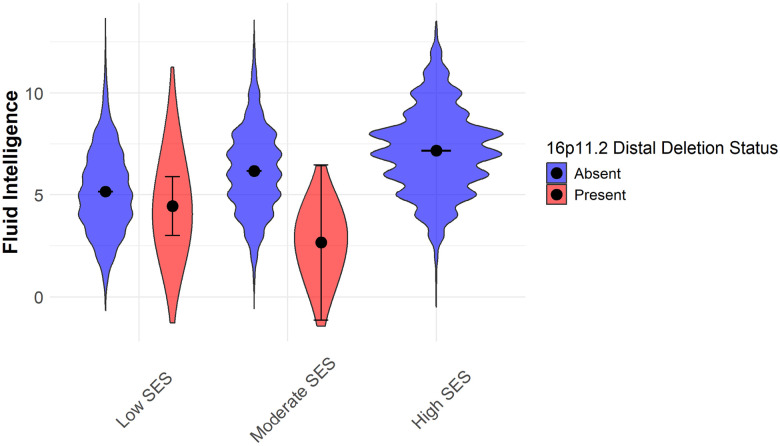
Interaction plot of 16p11.2 distal deletion status (present vs. absent) and socioeconomic status (SES) (low, moderate, high) on fluid intelligence. The x-axis indicates SES, while the y-axis shows mean fluid intelligence scores. Black points represent means, with error bars indicating confidence intervals. Blue colour represents individuals without the deletion (Absent), whereas the red colour represents those with the deletion (Present).

To examine how SES interacts with CNV carrier status in predicting FI, ANOVA models were conducted, incorporating interaction terms (Table S3). All SES variables demonstrated consistent and significant main effects across models (Fig. 1B, D). However, the impact of CNVs was more variable, showing specific SES × CNV interactions. After FDR correction, a significant interaction emerged between SES and the 16p11.2 distal deletion (F = 4.62, df = 1, p = 0.04; Fig. 1A). Among non-carriers, fluid intelligence showed a clear positive association with SES level. However, for 16p11.2 distal deletion carriers, FI scores were lowest in the moderate SES group, deviating from the expected trend. Notably, the absence of high-SES individuals among CNV carriers may have contributed to this pattern (Fig. 2).

Further post hoc comparisons revealed that CNV carriers in the moderate SES group exhibited the largest cognitive deficits, with FI scores significantly lower than both non-carriers in the same SES group and those in the high SES group. Unlike non-carriers, who displayed a steady increase in FI with the higher SES group, CNV carriers did not show the same benefit from increased SES.

**Figure 3:**
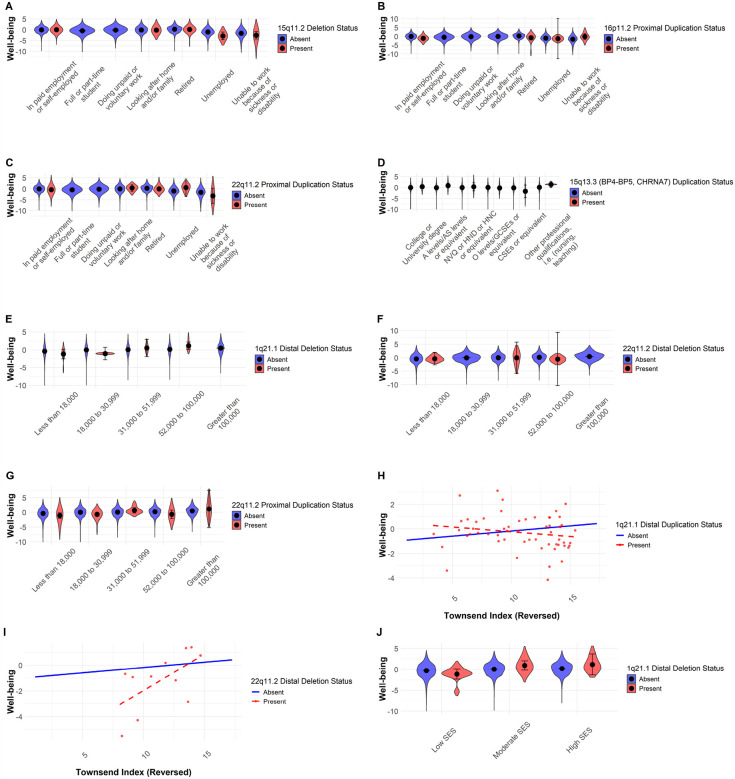
Interaction plot for the effect of the Copy Number Variants (CNVs) and socioeconomic status (SES) on well-being. The x-axis represents the SES variable, while the y-axis shows mean well-being scores. Violin plots show the data distribution for each group, with black points denoting mean values and error bars representing confidence intervals (A-G, J). Distribution dots are shown only for carriers for the Townsend deprivation index (reversed) (H, I). Blue colour represents individuals without the CNV (Absent), whereas the red colour represents those with the CNV (Present).

A similar pattern emerged for well-being (Fig. 1C, Table S4). Work status significantly interacted with CNV carrier status on well-being in the 22q11.2 proximal duplication (F = 3.54, df = 5, p < 0.001, Fig. 3C), 16p11.2 proximal duplication (F = 4.59, df = 4, p < 0.001, Fig. 3B), and 15q11.2 deletion (F = 3.15, df = 4, p = 0.02, Fig. 3A) models.

Post hoc analyses revealed that work status and well-being followed a linear trend in non-carriers, whereas CNV carriers exhibited greater variability, deviating from this expected trajectory. Income also interacted significantly with CNV carrier status, with notable effects for the 22q11.2 proximal duplication (F = 2.65, df = 4, p = 0.04, Fig. 3G), 22q11.2 distal deletion (F = 3.43, df = 3, p = 0.02, Fig. 3F), and 1q21.1 distal deletion (F = 3.21, df = 4, p = 0.02, Fig. 3E). While non-carriers demonstrated a linear relationship between income and well-being, CNV carriers displayed greater variability in outcomes.

The Townsend deprivation index (reversed), a measure of wealth, showed a trend toward lower well-being in CNV carriers. While significant interactions were detected for 22q11.2 distal deletion (F = 6.27, df = 1, p = 0.02, Fig. 3I) and 1q21.1 distal duplication (F = 7.12, df = 1, p = 0.01, Fig. 3H), post hoc comparisons were not significant (p = 0.138 and p = 0.070, respectively).

### Adulthood Stress and Childhood Stress

**Figure 4:**
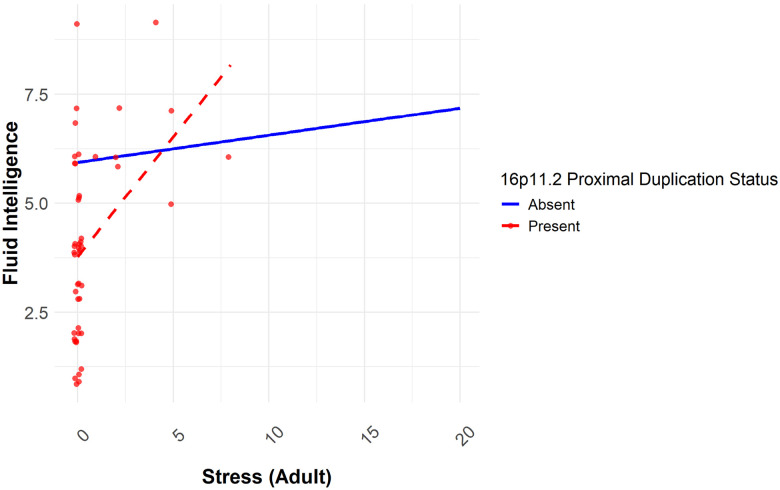
Interaction plot for the effect of the 16p11.2 proximal duplication and adulthood stress on fluid intelligence.

To investigate the role of stress in adulthood and childhood (AS and CS), interaction analyses were conducted for fluid intelligence and well-being (Table S5–S6). A significant interaction was found between 16p11.2 proximal duplication and AS (F = 6.76, df = 1, p < 0.01). While non-carriers maintained higher fluid intelligence across stress levels, carriers of the 16p11.2 proximal duplication showed a different trend: although their overall fluid intelligence was lower, it increased with rising stress (p < 0.001) (Fig. 4).

Main effects analysis revealed that both AS and CS consistently predicted fluid intelligence across models. Higher stress levels were associated with slightly better cognitive performance, indicating a small but robust positive effect independent of CNV status. In contrast, CNV effects were more variable and context-dependent, with smaller effect sizes and less consistent associations.

**Figure 5:**
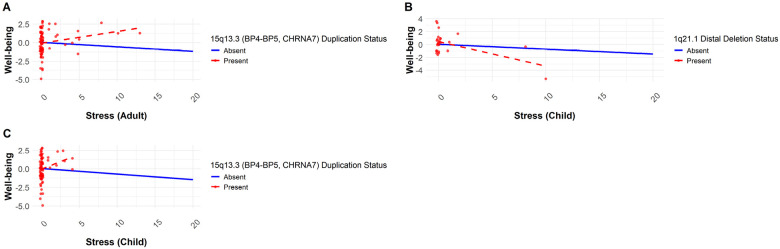
Interaction plot for the effect of the CNVs and stress variables on well-being. The x-axis represents the stress variable, while the y-axis shows mean well-being scores. The solid blue line represents individuals without the deletion (CNV absent), whereas the dashed red line represents those with the deletion (CNV present). Distribution dots are shown only for carriers.

Carriers of the 15q13.3 BP4–BP5 (*CHRNA7*) duplication exhibited higher well-being with increasing stress levels (F = 7.97, df = 1, p = 0.01 for AS; F = 5.28, df = 1, p = 0.03 for CS), whereas non-carriers experienced the expected decline. Post hoc analyses revealed no significant interactions between 15q13.3 BP4–BP5 (*CHRNA7*) duplication and AS or CS on well-being (p > 0.05). Even though 1q21.1 distal deletion carriers showed a significant interaction with CS (F = 6.58, df = 1, p = 0.01), post hoc was non-significant (p = 0.33), suggesting that stress does not affect the effects of these CNVs on well-being (Fig. 5).

The main effects for well-being mirrored those of fluid intelligence regarding stress, with stress in adulthood and childhood consistently showing significant associations across models.

### Lifestyle

We next examined the interaction between CNVs and lifestyle on fluid intelligence and well-being (Table S7–S8). Lifestyle emerged as a strong predictor of fluid intelligence, with healthier lifestyles generally associated with higher cognitive performance. However, significant CNV × lifestyle interactions were detected only for the 15q11.2 deletion (F = 3.60, df = 2, p = 0.04, Supplementary Fig. 1A) and 16p11.2 proximal deletion (F = 3.87, df = 2, p = 0.03, Supplementary Fig. 1B).

For 15q11.2 deletion carriers, lifestyle had a clear negative effect on fluid intelligence, particularly in the moderately healthy group, where FI was significantly lower than in all non-carrier groups (p < 0.01).

For 16p11.2 proximal deletion carriers, individuals in the least healthy group had the highest FI, though this finding was not statistically significant (p = 0.88), likely due to the large variability in the small sample size.

Carriers of the 22q11.2 distal duplication exhibited a significant interaction between lifestyle and well-being (F = 3.52, df = 2, p = 0.04) (Supplementary Fig. 2), suggesting that lifestyle influences well-being differently in carriers compared to non-carriers. Non-carriers exhibited stable well-being across lifestyle groups, whereas carriers showed a trend toward higher well-being in the least healthy group and lower well-being in healthier groups. However, post hoc analyses did not confirm significant differences between groups (p = 0.49).

Overall, lifestyle exerted a robust and consistent effect on well-being, while CNV-related effects varied considerably on specific gene–environment interactions.

## Discussion

This study investigated the interplay between CNVs and environmental factors: socioeconomic status, stress, and lifestyle, on fluid intelligence and well-being in the UK Biobank cohort. Our findings reveal that CNVs have variable, context-dependent effects on cognitive and psychological outcomes, underscoring the role of gene–environment interactions. SES, stress, and lifestyle were robust predictors of both traits, but their impact was often moderated by specific CNVs. While some results aligned with our hypothesis – that higher SES, lower stress, and healthier lifestyles would enhance outcomes – others deviated from expected patterns. Overall, environmental factors showed consistent associations regardless of CNV status, whereas CNV effects varied across environmental contexts. This highlights the specificity of CNV-related influences amid broader environmental trends.

The interaction between SES and the 16p11.2 distal deletion highlights the complexity of gene–environment interactions on cognitive outcomes. While higher SES is typically associated with improved fluid intelligence in non-carriers, this pattern does not hold for individuals with the 16p11.2 distal deletion. Moderate SES does not confer the same cognitive benefits to CNV carriers as it does to non-carriers, and in fact, appears to be the most detrimental SES category for these individuals. However, it is important to consider that the relatively high baseline SES in this predominantly middle-class cohort may have limited the variability in SES, potentially influencing these findings. CNVs contribute to structural brain differences that affect cognitive ability, which could subsequently impact SES status over time^31^. This highlights the need for longitudinal studies to determine whether SES primarily moderates CNV effects on cognition or if cognitive ability mediates the relationship between CNVs and SES disparities.

One possible explanation is that while SES generally provides cognitive advantages, individuals with 16p11.2 distal deletions may have biological constraints on cognitive adaptability, limiting the extent to which they benefit from enriched environments. Genes within the 16p11.2 distal region, such as *SH2B1* (involved in neurotrophic factor signaling^32–34^) and *RABEP2* (linked to vesicle trafficking^35^), may contribute to impaired plasticity or developmental constraints, limiting these individuals’ ability to fully benefit from the advantages of higher SES. Additionally, the absence of high-SES individuals among carriers further complicates interpretation, as it remains unclear whether a truly enriched environment might counteract these genetic constraints. This trend also aligns with the concept of “cognitive ceiling effects”, where genetic vulnerabilities impose an upper limit on potential cognitive gains. While non-carriers experience progressive increases in intelligence with SES, carriers may experience diminishing returns or even negative effects in certain SES contexts. This suggests that rare CNVs do not merely shift cognitive outcomes downward but may actively disrupt the mechanisms through which SES influences cognitive development^36,37^. Future research should investigate whether alternative protective factors, such as targeted educational interventions or cognitive training, might help mitigate the negative SES effects observed in CNV carriers.

Our analysis furthermore revealed a significant interaction between income and well-being in individuals with the 1q21.1 distal deletion. While higher income was associated with increased well-being across all groups, the pattern among CNV carriers was more variable, with some non-significant contrasts between income levels. Despite this variability, the trend suggests that income-related well-being benefits may be more pronounced for CNV carriers at the highest income levels. One potential explanation is that higher income provides greater access to healthcare, reduced financial stress, and improved living conditions, which could disproportionately benefit individuals with genetic vulnerabilities such as the 1q21.1 distal deletion. Furthermore, genes within the 1q21.1 locus, such as *BCL9* (involved in Wnt signaling and cognitive development^38^, and *NOTCH2NL*, crucial for neuronal differentiation in the brain^39^, may influence neurodevelopmental sensitivity to socioeconomic conditions. If individuals with the 1q21.1 deletion experience heightened stress responses due to underlying neurodevelopmental risk factors^40^, higher income might mitigate these effects, buffering against mental health risks associated with lower SES^41^. Similar interaction patterns were observed for both the 22q11.2 proximal duplication and distal deletion, where income moderated well-being more variably among CNV carriers than non-carriers. This pattern of findings reinforces the need to consider social context in understanding and mitigating the effects of genomic risk. However, not all post hoc comparisons were significant, particularly in the moderate-income range, where well-being improvements among CNV carriers did not differ significantly from those of non-carriers. This suggests that while higher income may provide some protective benefits, these effects are not universally stronger for CNV carriers across all income levels. Additionally, the wide confidence intervals for CNV carriers in the highest-income group indicate substantial variability, suggesting that other factors, such as medication, employment stability, or mental health interventions, may play a crucial role in determining well-being outcomes.

Our findings also reveal a significant interaction between work status and CNV carrier status on well-being, particularly in individuals with the 22q11.2 proximal duplication, 16p11.2 proximal duplication, and 15q11.2 deletion. While non-carriers followed a linear trend, where employment was generally associated with higher well-being, CNV carriers exhibited greater variability, deviating from this expected trajectory. For some, structured employment may provide stability and social engagement, improving well-being, while for others, job-related cognitive load and social demands may lead to increased stress and reduced well-being. The nonlinear effects observed suggest that work-related well-being outcomes in CNV carriers may be shaped by job type, workplace accommodations, and individual coping mechanisms.

Having examined SES-related moderators, we next turned to the role of stress as a key environmental factor. The interaction between adult stress and 16p11.2 proximal duplication reveals an intriguing pattern: while non-carriers show stable fluid intelligence across stress levels, carriers exhibit a paradoxical trend where increased stress is associated with higher fluid intelligence. However, it is crucial to recognize that carriers’ cognitive performance remains significantly lower than that of non-carriers across all stress levels. One possible explanation for this effect is that individuals with 16p11.2 proximal duplications may exhibit heightened neuroplasticity responses to stress, potentially using the stress-induced changes to enhance their cognitive performance. Stress-induced activation of pathways involving MAPK3^42^ and TAOK2^43^, both implicated in neuroplasticity and the MAPK/ERK signalling cascade may contribute to this adaptive response. Additionally, genes such as *SEZ6L2*^44^ and *DOC2A*^45^, which are critical for neuronal connectivity and neurotransmitter release, could moderate synaptic efficiency in response to environmental pressures, leading to temporary cognitive gains under moderate stress levels^46^. However, this apparent cognitive benefit does not imply an overall advantage, as carriers still demonstrated lower fluid intelligence scores than non-carriers. The small number of highly stressed carriers in our dataset may have exaggerated this trend, potentially inflating the observed interaction effect. Additionally, while short-term stress exposure might trigger compensatory neuroplastic changes, chronic stress could still have deleterious long-term consequences, such as emotional dysregulation or increased susceptibility to neuropsychiatric disorders. Further research is needed to clarify whether this effect represents true cognitive adaptation or a temporary compensatory response that may involve long-term cognitive costs.

Following the analysis of stress-related moderators, we finally investigated lifestyle factors as potential environmental influences on cognitive and well-being outcomes in CNV carriers. Our analysis revealed significant interactions between lifestyle and CNV status, particularly in the 15q11.2 region for fluid intelligence as confirmed by the post hoc tests. This deletion usually includes genes like *CYFIP1*^47^ and *NIPA1*^48^, which are implicated in synaptic plasticity and cognitive regulation. Attempting to adopt certain health behaviors may impose additional cognitive or physiological demands that carriers are less equipped to manage, leading to reduced cognitive performance. Unlike non-carriers, carriers may lack the neurodevelopmental resilience to benefit from partial lifestyle changes, resulting in a paradoxical decline in function under moderate lifestyle conditions. CNV carriers may thus require personalized lifestyle interventions that consider their neurodevelopmental and cognitive vulnerabilities.

Future longitudinal studies are needed to clarify causal relationships and the dynamic interplay between CNVs and environmental factors. Investigating pooled CNVs and aggregated environmental measures could reveal broader patterns shaping cognitive and psychological outcomes. Although our results suggest that CNVs moderate environmental effects, limited post hoc significance highlights the need for finer-grained analyses of within-group variability. Personalized strategies to enhance environmental support and reduce stress may still benefit CNV carriers, but more targeted research is required to guide effective interventions.

A key limitation of this study is the relatively small number of CNV carriers in the UK Biobank, which reduces statistical power, especially for rare variants. Additionally, the cohort’s composition: predominantly older, healthier, and self-selected individuals, may limit generalizability to clinical or more diverse populations. Given the cognitive challenges associated with CNVs, lower-functioning individuals may be underrepresented, and duplication carriers, who often present milder symptoms, may be overrepresented, introducing potential recruitment bias. Moreover, minimal exclusion criteria were applied to maximize sample size, which may have introduced unmeasured confounders such as undiagnosed neurological conditions. Despite these constraints, this study offers several strengths. It evaluates a broad range of CNVs, incorporates diverse environmental exposures, and applies a robust analytical framework. The inclusion of both CNV carriers and non-carriers facilitates direct comparisons, advancing our understanding of gene–environment interactions and CNV-related phenotypic variation.

This study provides novel insights into how CNVs interact with environmental factors: socioeconomic status, stress, and lifestyle, to shape fluid intelligence and well-being. Our findings highlight that CNVs contribute to cognitive and psychological vulnerabilities and moderate individual responses to environmental conditions. While higher SES, reduced stress, and healthier lifestyles generally promote well-being, these effects are not uniform in CNV carriers; some show paradoxical or nonlinear responses, underscoring the need for a more nuanced understanding of gene–environment interactions.

These results emphasize the importance of considering both genetic predispositions and environmental contexts when evaluating cognitive and psychological outcomes. Tailored interventions, such as educational support, stress management, and lifestyle modification, should reflect these interactions to better support CNV carriers. Longitudinal studies are needed to further clarify these dynamics, refine interventions, and improve outcomes for individuals with CNVs.

## Supplementary Material

This is a list of supplementary files associated with this preprint. Click to download.

- SupplementsUKB11.docx
- Supplements2.pdf

## Figures and Tables

**Table 1:** Number of carriers, controls, and excluded individuals for each analysis.

Copy Number Variant (CNV)	Carriers	Controls	Excluded
1q21.1 Distal Deletion	105	484,116	17,912
1q21.1 Distal Duplication	158		17,859
15q11.2 Deletion	1,341		16,676
15q11.2 Duplication	1,383		16,634
15q13.3 (BP4–BP5, *CHRNA7*) Deletion	38		17,979
15q13.3 (BP4–BP5, *CHRNA7*) Duplication	225		17,792
16p11.2 Proximal Deletion	125		17,892
16p11.2 Proximal Duplication	139		17,878
16p11.2 Distal Deletion	57		17,960
16p11.2 Distal Duplication	106		17,911
22q11.2 Distal Deletion	40		17,977
22q11.2 Distal Duplication	257		17,760
22q11.2 Proximal Deletion	10		18,007
22q11.2 Proximal Duplication	287		17,730
